# Factors contributing to the swimmer puppy syndrome found in Labrador retrievers

**DOI:** 10.1186/s12917-022-03226-3

**Published:** 2022-03-29

**Authors:** Mizuki Tomihari, Yuko Nobutoki, Nagachika Nakajima, Masashi Yanagawa, Michihito Tagawa, Koichi Hagiya, Tetsuro Nomura, Yoshinori Suwa, Hiroshi Suzuki

**Affiliations:** 1 Department of Clinical Veterinary Science, Obihiro University of Agriculture and Veterinary Medicine, Obihiro, Hokkaido 080-8555 Japan; 2 Department of Advanced Clinical Medicine, Division of Veterinary Science, Graduate School of Life and Environmental Sciences, Osaka Prefecture University, Izumisano, Osaka 598-8531 Japan; 3 Department of Life and Food Science, Obihiro University of Agriculture and Veterinary Medicine, Obihiro, Hokkaido 080-8555 Japan; 4 Department of Bioresource and Environmental Sciences, Faculty of Life Sciences, Kyoto Sangyo University, Kyoto, Kyoto 603-8555 Japan; 5 Hokkaido Guide Dogs for the Blind Association, Sapporo, Hokkaido 005-0030 Japan; 6 National Research Center for Protozoan Diseases, Obihiro University of Agriculture and Veterinary Medicine, Obihiro, Hokkaido 080-8555 Japan

**Keywords:** Swimmer puppy syndrome, Labrador retriever, Dog

## Abstract

**Background:**

Swimmer puppy syndrome is a disease found in neonatal puppies mainly characterized by the inability to stand, but its direct cause is unknown. Since swimmer puppies were observed infrequently but continuously among the Labrador retriever colony at the Hokkaido Guide Dogs for the Blind Association in Japan, based on their birth record and pedigree, factors related to the onset of swimmer puppy syndrome in Labrador retrievers were examined.

**Results:**

The total number of offspring over seven years was 436, of which 16 were swimmer puppies. Most of the affected puppies except one recovered steadily. As for the swimmer puppies, the litter size was significantly lower, and the body weights on the 10th and 28th day after delivery were significantly higher than the non-symptomatic puppies. These results suggested that the onset may be related to weight gain in the neonatal stages due to a small litter size. According to the genetic analysis, 26 ancestors common to the affected individuals were confirmed, but the causative individual could not be identified with the inbreeding coefficient. The heritability of the swimmer-puppy onset trait was 0.80, and the heritability for the the 10^th^-day body-weight trait was equally high at 0.78, both of which strongly suggest genetic involvement.

**Conclusions:**

In this study, the onset of swimmer puppy syndrome in the Labrador retrievers was associated with litter size and early weight gain, and result of study suggests that genetic influence might be involved.

## Background

Swimmer puppy syndrome is a disease found in dogs and cats during the neonatal period in which all four limbs are splayed laterally, and the main symptom is a ‘swimmer’-like movement [1], called so because the puppy shows difficulty in standing up and walking at the typical age (generally from the 2nd to the 4th week after birth), and there is a movement like rowing to the side while trying to walk around [1, 2]. It is also called flat-puppy syndrome. because the puppy spends most of its time in the prone position and often has flat breasts. It has also been reported that funnel chest is often present [1, 3].

Orthopedic, nutritional, neurological, genetic, and environmental factors have been discussed as causes of this syndrome [1, 2, 4–8], but the exact mechanisms of this syndrome are still unclear. Diagnostic criteria and general treatments have not been established, but treatments such as physiotherapy, bandages, and massage were suggested to yield good progress in some reports [2, 4–7, 9]. According to these reports, the prognosis varies depending on the severity, from cases leading to dyspnea and aspiration pneumonia to those that resolve themselves spontaneously.

Nganvongpanit et al*.* examined 2443 puppies for the relationship between the onset of swimmer puppy and the various factors, including breed, breed size, gender, litter size, and flooring types. Among them, they suggested that overweight due to a decrease in the litter number may be one of the risk factors [1]. However, because this report included many breeds and cases in various environments, the genetic or nutritional factors were still unknown.

On the other hand, at the Hokkaido Guide Dogs for the Blind Association in Japan, swimmer puppies have been seen every year continuously. Although symptoms of the affected individual varied, accurate records of the onset data were kept, and the pedigree could be followed.

Therefore, in this study, the risk factors of swimmer puppies found in Labrador retrievers maintained under the unified management protocol were examined. In addition, based on the pedigree information, the involvement of genetic factors was investigated based on the inbreeding coefficient and the BLUP animal model.

## Results

The total number of pups was 436. Of these, 16 were diagnosed as swimmer puppies, and 420 were non-symptomatic individuals (Table 1). The number of whelping, total number of pups, number of swimmer puppies, and the incidence rate are shown (Table 2). The 7-year incidence rate of swimmer puppies was 3.7%. Of the 16 swimmer puppies, one died from the portosystemic shunt at 52 days after birth. Other individuals began to stand up between the 17th and 70th days after birth (Table 1), and most of them showed an improving tendency about one month later with simple nursing care.

**Table 1:** List of swimmer puppies

	**Birth** **year**	**sire ID**	**dam ID**	**Sex**	**Litter** **size**	**Day of standing was first observed**	**Recovery to walk**	**Complication**
**swimmer 1**	2011	H200900081	H200500056	M	1	day 21	+	
**swimmer 2**	2011	H200800011	H200500044	F	1	day 70	+	
**swimmer 3**	2012	Q200700014	H200700080	F	1	day 28	+	
**swimmer 4**	2012	C200900024	H200600048	F	4	day 30	+	
**swimmer 5**	2013	H201100026	H201100062	F	2	day 39	+	
**swimmer 6**	2014	S201000009	H200800009	M	6	day 17	+	
**swimmer 7**	2014	H201200079	H200700045	F	5	day 55	+	
**swimmer 8**	2014	H201200079	H200700045	F	5	day 35	+	
**swimmer 9**	2014	H201200079	H200700045	M	5	day 35	+	
**swimmer 10**	2014	H201000065	H200700016	M	1	day 28	+	
**swimmer 11**	2014	T201000046	H200600048	F	6	day 32	+	
**swimmer 12**	2014	T201000046	H200600048	M	6	-	-	died on day 52 due to a portosystemic shunt
**swimmer 13**	2014	S201000009	H201000042	F	4	day 33	+	
**swimmer 14**	2014	S201000009	H201000042	M	4	day 33	+	
**swimmer 15**	2016	H201100057	H200800035	F	3	day 28	+	
**swimmer 16**	2016	H201100057	H200800035	F	3	day 28	+	

16 swimmer puppies are designated as swimmer 1 to swimmer 16. Individual identification numbers are shown for father dogs (sire ID) and mother dogs (dam ID)

**Table 2:** Swimmer puppy incidence by year

Birth year	Number of births	Total number of puppies	Total number of swimmer puppies	Incidence rate (%)
2011	18	92	2	2.2
2012	15	75	2	2.7
2013	10	41	1	2.4
2014	13	68	9	13.2
2015	13	73	0	0
2016	13	83	2	2.4
2017	1	4	0	0
total	83	436	16	3.7

In the swimmer puppy group, 6 were male and 10 were female (Table 1). In contrast, the non-symptomatic group had 220 were male and 200 were female. No statistically significant difference was found in the association with either breed/gender or onset (*p* > 0.1).

**Fig. 1:**
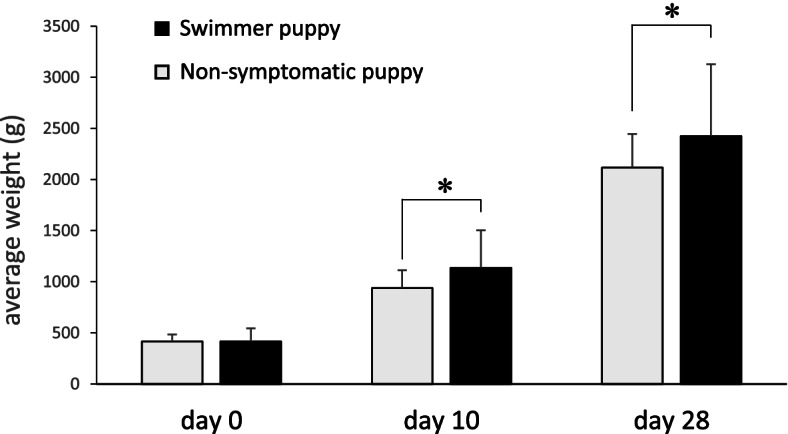
A comparison of the body weight at each day old between the swimmer puppy group (black, *n* = 16) and the non-symptomatic group (gray, *n* = 420). On the 10th and 28th day, the swimmer puppy group showed significantly higher values (* *P* < 0.01)

The average body weight of the swimmer puppy group was 416.6 ± 127.4 g at birth, 1135.0 ± 368.8 g on the 10th day, and 2424.8 ± 704.2 g on the 28th day. In contrast, the mean body weight of the non-symptomatic group was 416.3 ± 68.2 g at birth, 938.9 ± 172.6 g on the 10th day, and 2115.5 ± 330.7 g on the 28th day. A comparison of birth weight showed no significant difference between the two groups. On the other hand, the body weight on the 10th day and 28th day was significantly higher in the swimmer puppy group than in the non-symptomatic group (Fig. 1).

**Fig. 2:**
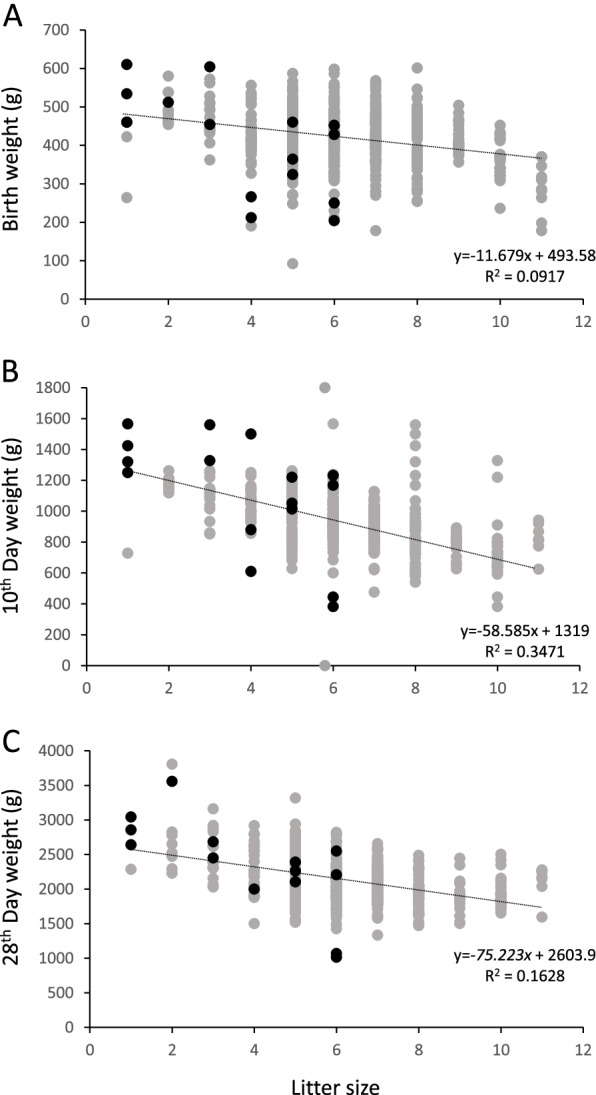
The correlation between the body weight and the number of litters. The swimmer puppy was indicated by a black circle (*n* = 16), and the non-symptomatic puppy was indicated by a gray circle (*n* = 420). The regression line for the body weight and the litter size was calculated and shown in the figure with the formula

The number of births during the seven years was 11 in the swimmer puppy group and 83 in the non-symptomatic group. The mean litter size was 3.1 ± 2.0 in the swimmer puppy group, significantly lower than 5.7 ± 1.8 in the non-symptomatic group (*p* < 0.01). In the distribution graph, the swimmer puppies tended to be in the region with low litter size and heavy body weight (upper left of the distribution map) compared to the non-symptomatic puppies (Fig. 2). The relationship between litter size and body weight in all individuals showed a negative correlation at birth, the 10th day, and the 28th day.

For 16 swimmer puppies, family trees were created according to the recorded pedigree. It could be traced back to up to 14 generations. As a result, there were 26 ancestors common to parents in all 16 affected individuals, which were in family lines that could be traced back to two individuals (× 000,000,073, × 000,000,290). Calculating the inbreeding coefficient, four ancestors showed high values in more than 10 of the swimmer puppies (Table 3). Of these, especially in × 000,000,073, high inbreeding coefficients were shown in all 16 swimmer puppies. However, when the inbreeding coefficient for four randomly selected non-symptomatic individuals were similarly calculated, some high values were also observed (Table 3). Then the posterior heritability for the swimmer puppy onset trait was calculated as 0.80 ± 0.10, and the heritability for the 10^th^-day body-weight trait was 0.78 ± 0.07. The posterior genetic correlation between these two traits was 0.13 ± 0.21, indicating a weak correlation (Table 4).

**Table 3:** Inbreeding coefficient derived from each ancestor

	** × 000,000,073**	** × 000,000,290**	** × 000,000,316**	** × 000,000,234**
swimmer 1	0.15	0	0.39	0.39
swimmer 2	0.21	0	0.05	0.05
swimmer 3	0.34	0.02	0	0
swimmer 4	0.29	0.02	0	0
swimmer 5	0.49	0	0	0
swimmer 6	0.04	0	0.21	0.21
swimmer 7–9	0.08	0.02	0.21	0.21
swimmer 10	0.28	0	0.05	0.05
swimmer 11,12	0.28	0.01	0.10	0.10
swimmer 13,14	0.04	0	0.20	0.20
swimmer 15,16	0.37	0.03	0.10	0.10
non-symptomatic 1	0	0	0	0
non-symptomatic 2	0.41	0	0.05	0.05
non-symptomatic 3	0.19	0.02	0.04	0.04
non-symptomatic 4	0.27	0	0.10	0.10

**Table 4:** Posterior heritability and genetic correlation between swimmer puppy onset traits and 10^th^-day body-weight traits

	**Genetic dispersal**	**Residual variance**	**Heritability**	**Genetic correlation of two traits**
**Swimmer puppy onset trait**	5.20 ± 4.43	0.99 ± 0.13	0.80 ± 0.10	0.13 ± 0.21
**10**^**th**^**-day body-weight trait**	16,769 ± 2344	4706 ± 1358	0.78 ± 0.07	

Data are expressed as mean ± posterior SD

## Discussions

The incidence rate of swimmer puppies in this study was 3.7%. The rate reported so far was 2.4% in Labrador retrievers, and 3.7% in golden retrievers in a study of puppies under 3 months of age who visited a Thai veterinary clinic [1]. Although it was not possible to simply compare them, the incidence rates were considered to be almost similar.

In the comparison of body weight at each period, swimmer puppies were showed significantly heavier than non-symptomatic puppies on the 10th and 28th days. On the other hand, the litter size, which had been suggested to be associated with the onset [1], was also shown to be significantly reduced in the swimmer puppy group. It was also reported that in dams with a small number of offspring, the properties of the milk were altered, and the calories also became high [10], which might lead to weight gain in puppies. In our results, negative correlations were found between the number of littermates and body weight in all individuals, indicating that the early weight gain due to the smaller litter size might have caused overweight and be involved in the onset. However, the causal relationship between the two had not been clarified, further investigation was needed for the elucidation.

Most of the affected individuals in this study had a good course except for one that died, and many of them stood up or walked in about one month. The Hokkaido Guide Dog Association had taken the following management protocol based on the knowledge so far. 1: Conduct intensive body-weight monitoring in individuals with small litter size and risk of overweight. 2: From the 10th to the 20th day after birth, if standing up was slower than other individuals or if flat breasts were observed, that puppy should be actively repositioned by turning it sideways. With these simple nursing care measures, many affected individuals gradually began to raise their upper body, chests became rounded, and they were able to stand up. This might be effective as a treatment for the early stage of swimmer puppies in retriever breeds.

It was possible to create a family tree based on a huge amount of kinship data and conduct a detailed examination of genetic factors. As a result of the inbreeding coefficient, a high value derived from × 000,000,073 was observed in 16 affected individuals. This suggested that × 000,000,073 might have a gene responsible for the development of swimmer puppy syndrome. However, even in the four randomly selected non-symptomatic individuals, some of them also showed a high value with respect to × 000,000,073. Since the Hokkaido Guide Dog Association had been breeding to maintain excellent pedigree, the ‘excellent’ × 000,000,073 was simply common to many offspring regardless of the onset. Therefore, it could not be strongly suggested that × 000,000,073 was the causative individual of the onset.

The posterior hereditability of the swimmer puppy onset trait showed a high value of 0.80 ± 0.10. It indicated that the onset of swimmer puppy was mostly influenced by heredity. Subsequently, the posterior heritability of the 10^th^-day body-weight trait also showed a high value of 0.78 ± 0.07. It is generally said that the heritability of growth-related factors tends to be high, and the heritability of mature body weight was already reported as 0.44 ± 0.07 in Labrador retrievers [11]. In addition, under the guidelines of the Hokkaido Guide Dog Association, the breeding environment of the mother dog was almost the same in all breeding volunteer households. Therefore, the environmental factor was relatively small compared to the genetic factor, and it might contribute to the high heritability of two traits. On the other hand, the genetic correlation between these two traits was poor at 0.13 ± 0.21. Although the details were unknown, it was possible that new other genetic factors were involved in the onset of swimmer puppy syndrome in retriever breeds.

In this study, it was not clarified what is the cause of swimmer puppy syndrome. Besides, histopathological information could not be obtained for many of the healed individuals. As a result, it was difficult to identify the affected area and comprehensively analyze it. In swimmer puppies, the changes in neuromuscular synaptic function, inappropriate myelination, poor development of peripheral motor neurons, and delayed muscle development were highlighted in previous reports [3, 8]. It has also been suggested that the other genetic factors are involved in locomotive abnormalities. To elucidate the detailed pathogenesis of this syndrome, further studies are needed in the future.

## Conclusions

This study revealed that the litter size and weight gain might be involved in the onset of swimmer puppy syndrome found in one breed dogs, Labrador retrievers. Most swimmer puppies could walk by simple nursing care at early stage. In addition, it was considered that the genetic influence on the onset was extremely high. Although the cause of swimmer puppy syndrome in Labrador retrievers still remains unclear, it is expected that this syndrome will be elucidated by further detailed analysis in the future.

## Methods

### Dogs

Detailed postnatal records among the puppies born between 2011 and 2017 were used in the Labrador retriever breeding colony at the Hokkaido Guide Dogs for the Blind Association in Japan. Their pedigree data kept by the AGBN (Asia Guide Dog Breeding Network) was used for genetic analysis. It was excluded from this study for the dogs which could not be tracked with accurate postnatal records.

## Inclusion criteria for swimmer puppy

Based on the inspection data by the veterinarian in charge of the Guide Dog Association, a swimmer puppy was defined as followed. Dogs that were seen to have all the following three symptoms at the same time between the 14th and 28th days after birth: 1) the forelimbs, hind limbs, or all four limbs are laterally deployed, 2) flat chest, 3) unable to stand. All individuals were inspected by the same veterinarian.

## Data Analysis

Data were collected for incidence rate, prognosis, breed, sex, weight, and the litter size. The incidence rate of swimmer puppy was expressed as a percentage by dividing the number of affected individuals by all births. The prognosis survey was traced back with all postnatal data. The litter size, and body weight records at birth, 10 days, and 28 days after birth were used and compared between litters where swimmer puppy appeared (swimmer puppy group) and the litters without swimmer puppy (non-symptomatic group). The correlation between the litter size and the body weight at each period was statistically analyzed.

## Inbreeding coefficient

The inbreeding coefficient was calculated based on the assumption that the onset of swimmer puppy is a qualitative trait and a hereditary disease due to a single recessive gene. An ancestor that frequently appears among the blood relatives of the affected individual was identified, and when that individual was designated as the common ancestor (A), the inbreeding coefficient derived from A was calculated for all individuals with records using the following formula.

$${F}_{{X}_{A}}=\sum {\left(\frac{1}{2}\right)}^{{n}_{iA}}\left(1+{F}_{A}\right)$$

where ${F}_{{X}_{A}}$ is the inbreeding coefficient of the individual X derived from A, ${n}_{iA}$ is the number of individuals on the i-th pathway that connects one parent to the other parent via A, and ${F}_{A}$ is the common ancestor A's inbreeding coefficient. ${F}_{{X}_{A}}$ was calculated for all common ancestors appearing among the pedigree The values were estimated using the program inbh (http://www.obihiro.ac.jp/~suzukim/masuda/software_inbcoef.html), which applied the method of Meuwissen and Luo [12]. The program was modified for estimating the inbreeding coefficient originated by common ancestor (A). In order to search for the causative common ancestor, individuals with golden parents were excluded from consideration, and only Labradors and hybrids were included.

## Heritability and genetic correlation

Assuming that the onset of swimmer puppy is a quantitative trait, the heritability was calculated. Heritability is a measure of how much an individual's phenotypic value is determined by additive genetic effects. A total of 436 records with 10,946 individuals in pedigree records were used to estimate genetic parameters of Labradors. The onset trait of swimmer puppy in retriever species was estimated using the best linear unbiased prediction (BLUP) animal model below [13, 14].

$$\mathrm{Formula }1: \mathcal{l}=S+L+u+e$$

$$\mathrm{Formula }2:y=S+L+u+\mathrm{e}$$

where ℓ is the liability records expressed by 0 (normal) or 1 (onset) for the onset of swimmer puppy, S is the fixed effect of gender (2 levels), and L is the covariate of the number of litters, u is the additive genetic effect, and e is the residual effect. The body weight on the 10th day was also regarded as a phenotype, and the heritability was calculated in the same manner (Formula 2). Where $y$ is observation of body weight, and $S$, $L$, $u$ and $\mathrm{e}$ are the same as in Formula 1. The genetic correlation between the above two traits was estimated according to the bivariate BLUP animal model analysis. The THRGIBBSf90 program (http://nce.ads.uga.edu/~ignacy), which could handle threshold traits, was used to estimate variance components and best linear unbiased estimators (BLUE) and BLUP [13]. Then Heritability h^2^ was shown as additive genetic variance (A) / additive genetic variance + residual variance (A + R). To calculate the posterior means and standard deviations of (co)variances, heritabilities and genetic correlation, 400 000 samples after a burn-in of 100 000 iterations were used. Convergence was determined by using visual inspection of the plots of Gibbs samples.

## Statistical analysis

EZR (EazyR: https://www.jichi.ac.jp/saitama-sct/SaitamaHP.files/statmed.html) was used for statistical analysis, and the chi-square test and Mann–Whitney U test were performed as statistical processing.

## Data Availability

The data containing information on common ancestry that support the findings of this study are available from the corresponding author on reasonable request and with permission of Hokkaido Guide Dogs for the Blind Association in Japan.

## Abbreviations

AGBN: Asia Guide Dog Breeding Network
BLUP: Best Linear Unbiased Prediction
BLUE: Best Linear Unbiased Estimators
EZR: EazyR
